# Effectiveness of triple therapy with dapagliflozin add-on to dual therapy over 52 weeks in patients with uncontrolled type 2 diabetes mellitus in a centre of high complexity, Cali-Colombia

**DOI:** 10.20945/2359-3997000000319

**Published:** 2021-01-14

**Authors:** Vanessa Bedoya Joaqui, Nathalia Buitrago Gómez, Reinaldo Carvajal Ortiz, Luis Miguel Osorio Toro, Jenny Patricia Muñoz Lombo, Carlos Alberto Salgado Cifuentes, Mónica Andrea Morales García, Alín Abreu Lomba

**Affiliations:** 1 Universidad Libre Departamento de Medicina Interna GIMI1 (Grupo Interinstitucional de Medicina Interna 1) Cali Colombia Departamento de Medicina Interna, GIMI1 (Grupo Interinstitucional de Medicina Interna 1), Universidad Libre, Cali, Colombia; 2 Centro Médico Imbanaco S.A. Cali Colombia Centro Médico Imbanaco S.A., Cali, Colombia; Departamento de Medicina Interna, GIMI1 (Grupo Interinstitucional de Medicina Interna 1), Universidad Libre, Cali, Colombia; Universidad Libre Departamento de Medicina Interna GIMI1 (Grupo Interinstitucional de Medicina Interna 1) Cali Colombia; 3 Universidad Santiago de Cali Departamento de Medicina Interna Cali Colombia Departamento de Medicina Interna, Universidad Santiago de Cali, Cali, Colombia; 4 Centro Médico Imbanaco S.A. Cali Colombia Centro Médico Imbanaco S.A., Cali, Colombia

**Keywords:** Diabetes mellitus, combined modality therapy, hypoglycemic agents

## Abstract

**Objectives:**

To evaluate the effectiveness of adding dapagliflozin as an intensification strategy for the treatment of patients with uncontrolled type 2 diabetes mellitus (T2DM).

**Materials and methods:**

A historical cohort study was conducted in 123 adult patients over 18 years old who were diagnosed with uncontrolled T2DM, who received dapagliflozin add-on to their dual base treatment: metformin plus glibenclamide (n = 32), metformin plus saxagliptin (n = 29), metformin plus exenatide (n = 28), or metformin plus insulin (n = 34). The endpoints were evaluated using analysis of variance.

**Results:**

All the patients completed a 52-week follow-up. Overall, 52.85% of patients were female, the Hispanic population represented the largest proportion of patients in all groups (60.98%), and the mean ± SD patient age and body weight were 55.05 ± 7.58 years and 83.55 ± 9.65 kg, respectively. The mean ± SD duration of T2DM, glycated hemoglobin (HbA_1c_), and fasting plasma glucose (FPG) were 5.93 ± 2.98 years, 8.1 ± 0.53%, and 166.03 ± 26.80 mg/dL, respectively. The grand mean changes of HbA_1c_, FPG, body weight and blood pressure showed a decreasing trend during the study period and it was statistically significant in all groups (p-value = <0.001). The proportion of patients achieving HbA_1c_ target (<7%) was highest in the group that used a dapagliflozin add-on to metformin plus saxagliptin.

**Conclusion:**

The addition of dapagliflozin as an alternative for intensification of dual therapy consistently improved, not only FPG and HbA_1c_, but also body weight and blood pressure, with statistically significant results.

## INTRODUCTION

The ongoing epidemic of diabetes mellitus and its complications constitutes a global health threat. In 2019, the International Federation of Diabetes estimated that 9.3% of the global adult population between the ages of 20 and 79 lives with diabetes and predicted an increase of 51% by 2045 (approximately 10.9% or 700 million adults), with Central and South America contributing 7% of all cases (1). In addition, the CARMELA study found a general prevalence of diabetes in Latin America of 7%; in Colombia, specifically in Bogotá, the prevalence was 7.4% in men and 8.7% in women (2).

Type 2 diabetes mellitus (T2DM) contributes extensively to mortality and disability worldwide, and the increased prevalence of childhood obesity in many countries has contributed to an increase in T2DM in the pediatric population, which tends to predispose individuals to the development of complications in early adulthood (3). Complications of T2DM are quite common. The IMPROVE study, which was conducted in eight countries and involved more than 50,000 patients, reported a high prevalence of complications: half of the patients suffer from microvascular complications, and 28% experienced macrovascular complications(4). The International Diabetes Management Practice Study (IDMPS) also reported high microvascular and macrovascular complications rates at 55.3% and 26.1%, respectively (5).

Effective T2DM management requires sustained glycemic control to reduce the risk of such complications, as shown by the UKPDS, in which, for each 1% reduction in glycated hemoglobin (HbA_1c_), acute myocardial infarctions decreased by 14%, microvascular complications decreased by 37%, and diabetes related deaths decreased by 21% (6). However, achieving the therapeutic objective of HbA_1c_ is difficult in a significant percentage of patients. In a retrospective study aimed at assessing the quality and effectiveness of diabetes care for a group of patients in Colombia, only 6.9% of patients achieved the goals recommended for control of glucose, lipids, and blood pressure (BP) (7).

The 2020 guidelines of the American Diabetes Association (ADA) recommend a patient-centered approach that considers various pharmacological agents, as well as the risk of hypoglycemia, comorbidities, impact on weight, cost, adverse events risk, and patients’ preferences (8). In the treatment algorithm, among the options for glycemic control, the drug of choice is metformin if it is tolerated and not contraindicated. The initiation of dual therapy should be considered in those patients recently diagnosed with T2DM who have HbA_1c_ greater than or equal to 1.5% (12.5 mmol/L) above their glycemic target; upgrading to triple therapy is recommended if adequate control is not achieved after 3 months (9,10). However, when glycemic control is not achieved, there is a range of therapeutic possibilities exist, including reversible selective sodium and glucose co-transporter 2 (SGLT2i) inhibitors, a class of drugs that inhibit glucose reabsorption in the renal proximal tubule, with a consequent glucosuric effect that decreases HbA_1c_, weight, and systolic blood pressure (BP) (11,12).The use of these drugs in monotherapy and combination therapy produces a complementary effect that addresses several of the pathological phenomena that occur in T2DM (13).

The ADA’s 2020 guidelines are quite emphatic in mentioning the importance of intensifying treatment immediately in those patients with T2DM who do not achieve their therapeutic goals (10). The American Association of Clinical Endocrinologists (AACE), in its 2020 algorithm for glycemic control, made the SGLT2i the second option in the hierarchical order of choice when initiating triple therapy and even in dual therapy after GLP1 analogues (14).

To the best of our knowledge, this is the first study at the national and Latin American level that compares the effectiveness of 4 triple therapy treatment groups in patients with uncontrolled T2DM. The aim of this study is to evaluate the effectiveness of adding dapagliflozin as an intensification strategy for the treatment of patients with uncontrolled T2DM.

## Materials and Methods

### Study design

A historical cohort study was conducted between August 2015 and September 2018, in patients over the age of 18 who were diagnosed with uncontrolled T2DM and received follow-up care at the Endocrinology Unit of Centro Médico Imbanaco S.A. in Cali - Colombia. The study protocol was approved by the Ethics Committee at Centro Médico Imbanaco S.A. in Cali, Colombia, and the study was conducted in accordance with the Declaration of Helsinki and the International Conference on Harmonization/Good Clinical Practice Guidelines. All patients were fully informed of the characteristics and purpose of the present study, and gave their informed consent.

### Participants

Patients were eligible for inclusion in the study if they fulfilled the following criteria: 1) female or male aged ≥ 18 years; 2) diagnosis of T2DM, defined as: prior documentation of T2DM based on ADA criteria such as fasting plasma glucose (FPG) > 126 mg/dL or HbA1c ≥ 6.5% or 2-h plasma glucose ≥ 200 mg/dL during an oral glucose tolerance test, or random plasma glucose ≥ 200 mg/dL in patients with classic symptoms of hyperglycemia or hyperglycemic crisis. 3) inadequate glycemic control (HbA_1c_ > 7%), despite at least 3 months of dual therapy that included a stable dose of metformin ≥ 1700 mg/day in combination with one of the following anti-hyperglycemic medications: glibenclamide 10 mg/day, saxagliptin 5 mg/day, exenatide 2 mg/week, or any insulin before enrollment and follow-up by the endocrinology specialist.

Exclusion criteria included recent use (within 3 months) of any other anti-hyperglycemic medications, chronic cystitis and/or recurrent urinary tract infections (3 or more in the last year), a systolic blood pressure of less than 90 mmHg, an estimated glomerular filtration rate below 45 ml per minute per 1.73 m^2^ of body-surface estimated using the CKD-EPI equation, and refusal to consent to participate in the study.

### Study groups

The patients were divided into 4 groups according to their basic dual therapy: metformin plus glibenclamide 10 mg/day orally; metformin plus saxagliptin 5 mg/day orally; metformin plus exenatide 2 mg/week subcutaneously; and metformin plus insulin. Patients were grouped according to the drug used most frequently at the time the study began. Patients who received any combination other than the ones described above were excluded. Dapagliflozin 10 mg/day orally was added as a third drug in all groups, as an intensification alternative.

Patients received suggestions regarding modifications to their lifestyle, such as reducing their intake of carbohydrates, saturated fat, red meat, sweets, and sugar-sweetened beverages and increasing their intake of nutrient-dense foods such as vegetables, fruits, low-fat dairy products, lean meats, fish and other seafood, beans, nuts, seeds, and whole grains. In addition, it was recommended that they complete 150 minutes or more of moderate-to-vigorous aerobic activity per week, spread over at least 3 days, with no more than 2 consecutive days without activity, as well as 2-3 sessions per week of exercise resistance on non-consecutive days. During the medical follow-up visit, the specialist verified that they complied with the recommendations.

### Variables

We collected demographic and clinical variables such as HbA_1c_ concentration, fasting plasma glucose (FPG) concentration, body weight, and systolic and diastolic BP. The clinical variables were recorded at the beginning of the study, and at 12, 24, and 52 weeks.

### End Point Assessment

The primary endpoint was the mean change in HbA_1c_, from baseline to week 52. Secondary endpoints regarding glycemic control included the proportion of patients who reached an HbA_1c_ target of less than 7% by week 52, and the mean change in FPG from baseline to week 52. Secondary endpoints in the assessment of cardiovascular risk factors included the change in systolic and diastolic BP and change in body weight from the beginning of the study to week 52.

### Safety Assessment

Safety was assessed by collecting data from medical history records concerning adverse events such as hypoglycemia, hypotension, and urinary tract infections. Hypoglycemia was classified as minor (symptomatic or asymptomatic with plasma glucose concentration of < 63 mg/dL, regardless of the need for external assistance) or major (symptomatic requiring third-party assistance because of severe impairment in consciousness or behavior with or without a plasma glucose concentration of < 54 mg/dL), and hypotension was defined as a low BP (less than 90/60mmHg).

### Statistical analysis

The information was collected in a Windows Excel database (Microsoft, 2016) and analyzed using SPSS20.0 (IBM-SPSS Inc., Chicago, IL). Demographic and clinical characteristics were described using means, standard deviation and prevalence. The Shapiro-Wilk test was used to compare the statistical normality of the endpoints, which were evaluated using analysis of variance for repeated measurements to contrast the mean values at each moment in time and the grand mean at week 52. The Bonferroni post-hoc test for multiple comparisons was used for pairwise comparison. The assumption of variance homogeneity was verified based on the sphericity test. In case of non-compliance, the Greenhouse-Geisser statistic was used. An analysis was completed to determine which of three models (linear, quadratic or cubic) best suited the effectiveness of triple therapy, based on the sum of type III squares and the p-value obtained. A level of statistical significance of α = 0.05 was established a priori. Correlations among variables were measured by coefficient of determination and repeated measures on general linear models.

## Results

### Patients

The first patient was enrolled on August 3, 2015, and the last completed the study on September 28, 2018. Of the 123 patients enrolled, 100% completed the 52-week follow-up. Overall, 52.85% of patients were female, the Hispanic population represented the largest proportion of patients in all groups, and 75.6% had a history of high BP. The mean ± SD patient age, duration of T2DM, HbA1c, FPG, body weight, systolic BP and diastolic BP were 55.05 ± 7.58 years, 5.93 ± 2.98 years, 8.1 ± 0.53%, 166.03 ± 26.80 mg/dL, 83.55 ± 9.65 kg, 148.2 ± 19.3 mmHg, and 87.1 ± 7.5 mmHg, respectively.

All patients with a history of arterial hypertension received at least one antihypertensive medication. Of these, 23.7% received pharmacological management in monotherapy with angiotensin-converting-enzyme inhibitors (ACE) inhibitors, angiotensin II receptor blockers (ARBs) or dihydropyridine (DHP) calcium-channel blockers (CCBs); 60.2% received dual antihypertensive treatment based on ARBs plus thiazide diuretics or DHP CCBs, and 16.1% received 3 or more antihypertensive medications.

At the beginning of the study, statistically significant differences were observed in FPG (p-value = 0.019) and in the duration of T2DM (p-value = 0.020) among the four groups. The patients in the dapagliflozin add-on to metformin plus saxagliptin group had been diagnosed with diabetes longer (7.3 ± 2.9 years) and had a higher FPG concentration levels (173.5 ± 23.2 mg/dL) when compared to the other three groups. No statistically significant differences were observed in the other demographic and clinical variables at baseline (Table 1).

**Table 1:** Demographic and baseline characteristics

Characteristics	Dapagliflozin add-on to metformin plus glibenclamide (n = 32)	Dapagliflozin add-on to metformin plus saxagliptin (n = 29)	Dapagliflozin add-on to metformin plus exenatide (n = 28)	Dapagliflozin add-on to metformin plus insulin (n = 34)	p value
Age, years	52.8 ± 9.2	55 ± 8.4	55 ± 6.8	57.4 ± 5.9	0.123
Sex, n (%)					0.410
Female	59.4 (19)	41.4 (12)	60.7 (17)	50 (17)	
Male	40.6 (13)	58.6 (17)	39.3 (11)	50 (17)	
Race, n (%)					0.731
Hispanic	68.7 (22)	55.2 (16)	60.7 (17)	58.8 (20)	
Afro-descendant	31.2 (10)	44.8 (13)	39.3 (11)	41.2 (14)	
Weight, kg	83.2 ± 9.9	80.7 ± 8.7	86.4 ± 9.6	83.9 ± 10.4	0.189
HbA_1c_, (%)					0.964
Mean	8 ± 0.5	8.1 ± 0.6	8 ± 0.5	8.3 ± 0.5	
Median	8.0	7.9	8.1	8.4	
FPG, mg/dL	170.3 ± 49.5	173.5 ± 23.2	168.7 ± 17.0	151.6 ± 17.5	0.019*
Duration of T2DM, years	5.3 ± 3.1	7.3 ± 2.9	4.5 ± 2.7	6.6 ± 3.2	0.020*
History of high BP (%)	68.8 (22)	86.2 (25)	75 (21)	73.5 (25)	0.446
Systolic BP, mmHg	149.2 ± 22.9	154.2 ± 21.9	143.1 ± 17.6	146.1 ± 14.6	0.167
Diastolic BP, mmHg	86.1 ± 7.4	90.1 ± 6.3	85.5 ± 8.1	86.5 ± 8.0	0.085

Data are mean (±SD) or n (%). HbA_1c_: glycated hemoglobin; FPG: fasting plasma glucose; T2DM: type 2 diabetes mellitus; BD: blood pressure.

* Statistically significant differences.

### Effectiveness

#### Primary effectiveness variable

The criteria for assessing both primary and secondary effectiveness endpoints are shown in Table 2. The primary endpoint was the mean change in HbA_1c_, values during the study period in uncontrolled diabetic patients during the study period; this showed a linear downward trend from baseline to week 52. The change was observed between the treatment groups starting in week 12 and continued until the end of the study (p-value = <0.001). Changes in HbA1c at week 52 were significantly greater with dapagliflozin add-on to metformin plus insulin. The difference (95% CI) in grand mean change from baseline in HbA_1c_ was -0.92 (-0.80 to -1.0); p-value= <0.00, than with either dapagliflozin add-on to metformin plus saxagliptin, exenatide, or glibenclamide, for which the differences (95% CI) in grand mean change were -0.73 (-0.59 to -0.81), -0.70 (-0.58 to -0.82) and -0.65 (-0.50 to -0.70), respectively. The final mean HbA1c values in each group were 7.2 ± 0.4%, 7.1 ± 0.5%, 7.0 ± 0.4%, and 7.2 ± 0.4%, respectively.

**Table 2:** Grand mean change from baseline at 52 weeks for primary and secondary effectiveness endpoints

	Dapagliflozin add-on to metformin plus glibenclamide (n = 32)	Dapagliflozin add-on to metformin plus saxagliptin (n = 29)	Dapagliflozin add-on to metformin plus exenatide (n = 28)	Dapagliflozin add-on to metformin plus insulin (n = 34)
**HbA_1c_, %**				
Baseline	8.0 ± 0.5	8.1 ± 0.6	8.0 ± 0.5	8.3 ± 0.5
Week 12	7.7 ± 1.5	7.6 ± 0.6	7.6 ± 0.4	7.7 ± 0.5
Week 24	7.2 ± 0.5	7.2 ± 0.6	7.3 ± 0.4	7.2 ± 0.4
Week 52	7.2 ± 0.4	7.1 ± 0.5	7.0 ± 0.4	7.2 ± 0.4
Change, grand mean (IC 95%) at week 52	-0.65 (-0.50 to -0.70); p < 0.001	-0.73 (-0.59 to -0.81); p < 0.001	-0.70 (-0.58 to -0.82); p < 0.001	-0.92 (-0.80 to -1.0); p < 0.001
**FPG, mg/dL**				
Baseline	170.3 ± 49.5	173.5 ± 23.2	168.7 ±17.0	151.6 ± 17.5
Week 12	150.6 ± 30.5	147.2 ± 18.8	150.7 ± 26.4	138.4 ± 11.8
Week 24	129.1 ± 29.4	123.6 ± 27.2	136.1 ± 18.4	127.7 ± 16.3
Week 52	121.4 ± 28.1	111.1 ± 22.1	127.8 ± 22.0	120.7 ± 20.8
Change, grand mean (IC 95%) at week 52	-36.63 (-31.38 to -41.88); p < 0.001	-46.20 (-39.22 to -53.19); p < 0.001	-30.50 (-25.98 to -35.02); p < 0.001	-22.67 (-19.67 to -25.67); p < 0.001
**Weight, kg**				
Baseline	83.2 ± 9.9	80.7 ± 8.7	86.4 ± 9.6	83.9 ± 10.4
Week 12	82.1 ± 9.7	79.8 ± 8.4	84.2 ± 9.4	83.0 ± 10.2
Week 24	81.8 ± 9.6	79.2 ± 8.5	83.2 ± 9.6	82.3 ± 10.3
Week 52	81.6 ± 9.6	78.7 ± 8.4	82.6 ± 9.4	82.1 ± 9.9
Change, grand mean (95% IC) at week 52	-1.36 (-1.27 to -1.45); p < 0.001	-1.52 (-1.31 to -1.73); p < 0.001	-3.04 (-2.73 to -3.36); p < 0.001	-1.39 (-1.24 to -1.54); p < 0.001
**Systolic BD, mmHg**				
Baseline	149.2 ± 22.9	154.2 ± 21.9	143.1 ± 17.6	146.1 ± 14.6
Week 12	135.9 ± 16.6	135.8 ± 15.4	128.9 ± 26.7	142.7 ± 12.7
Week 24	132.0 ± 15.6	128.7 ± 13.9	127.5 ± 8.3	134.7 ± 12.7
Week 52	130.4 ± 11.8	126.8 ± 15.7	124.1 ± 8.3	130.3 ± 10.0
Change, grand mean (95% CI) at week 52	-16.48 (-15.05 to -17.46); p < 0.001	-23.82 (-22.02 to -25.62); p < 0.001	-16.31 (-15.36 to -17.26); p < 0.001	-10.24 (-8.13 to -12.35); p < 0.001
**Diastolic BD, mmHg**				
Baseline	86.1 ± 7.4	90.1 ± 6.3	85.5 ± 8.1	86.5 ± 8.0
Week 12	84.5 ± 7.4	85.3 ± 6.7	82.9 ± 7.7	84.2 ± 7.5
Week 24	83.3 ± 7.3	82.4 ± 7.2	81.6 ± 5.9	83.4 ± 7.1
Week 52	82.2 ± 7.1	79.2 ± 7.4	81.0 ± 5.8	81.5 ± 7.0
Change, grand mean (95% IC) at week 52	-2.82 (-2.42 to -3.22); p < 0.001	-7.77 (-6.61 to -9.93); p < 0.001	-3.63 (-3.26 to -4.0); p < 0.001	-3.45 (-2.99 to -3.91); p < 0.001

Data are mean (±SD). Grand mean is the mean of the means of several subsamples [95% confidence interval (CI)]. HbA_1c_: glycated hemoglobin; FPG: fasting plasma glucose; BD: blood pressure.

### Secondary effectiveness variable

#### Glycemic control

**Figure 1:**
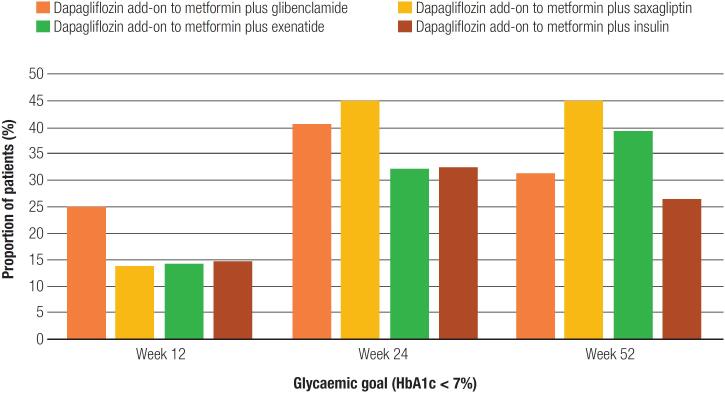
Proportion of patients with HbA1c < 7% (%).

The group that used dapagliflozin as an add-on therapy to metformin plus insulin achieved a greater reduction in HbA_1c_, but the proportion of patients achieving HbA_1c_ target (<7%) was highest in those who used dapagliflozin as an add-on to metformin plus saxagliptin in a sustained manner starting in week 24 (Figure 1). Regarding the glycemic control included in the mean change in FPG from baseline to week 52, a linear downward trend was observed for all groups from the earliest time point assessed (p-value= <0.001). Reductions in FPG from baseline were significantly greater with dapagliflozin add-on to metformin plus saxagliptin [at week 52, the difference (95% CI) in grand mean change was -46.20 (-39.22 to -53.19); p-value= <0.00]. Overall, only 26% of patients achieved the HbA_1c_ target of <7%; however, 43.1% of the patients reported a reduction of >1% in their basal HbA_1c_.

A greater downward trend was observed among men and elderly patients, but without statistically significant differences. Other variables evaluated, such as the evolution of T2DM diagnosis, weight loss and race, did not show the same trend.

#### Insulin dose

The insulin dose was not modified significantly throughout the study. Table 3 shows insulin dose at baseline and weeks 12, 24, and 52 (p-value= 0.9023).

**Table 3:** Insulin dose

	Baseline	Week 12	Week 24	Week 52
Minimum (IU/day)	8	10	8	8
Maximum (IU/day)	27	30	32	32
Mean (IU/day)	18.6	18.2	17.8	18.4
Standard deviation (IU/day)	5.5	5.7	6.8	7.7

IU: international units.

#### Cardiovascular risk factors

The secondary endpoints in the assessment of cardiovascular risk factors were the change in systolic and diastolic BP and change in body weight from the beginning of the study until week 52. Reductions in systolic and diastolic BP from baseline were significantly greater and earlier in patients who used dapagliflozin as an add-on to metformin plus saxagliptin [at week 52, the differences (95% CI) in grand mean change was -23.82 (-22.02 to -25.62); p-value <0.001 and -7.77 (-6.61 to -9.93); p-value <0.001, respectively. In the groups that used dapagliflozin as an add-on to metformin plus glibenclamide, exenatide, and insulin, even though a linear downward trend was observed from the beginning, the differences were statistically significant from week 24 (p-value = <0.001).

The reduction in systolic BP was greater among men, elderly individuals, patients of African descent, and those who received a β-blocker or calcium-channel blocker as part of their antihypertensive treatment, but the differences were not statistically significant. Other evaluated variables, such as the evolution of T2DM diagnosis, did not show the same trend. Weight loss was not related to a reduction in systolic BP (R2-value: 0.03, p-value:0.067).

A significant reduction in weight was observed in all the study groups, and it was statistically significant for all measurements (p-value <0.001), maintaining a linear downward trend in all groups. The mean changes from baseline in weight was greater at each point in time with dapagliflozin add-on to metformin plus exenatide [adjusted mean ± SE change at week 12 (−2.18 ± 1.66 kg), at week 24 (−3.14 ± 1.78 kg) and at week 52 (−3.79 ± 1.81 kg)]. At week 52 the differences (95% CI) in grand mean change from baseline in HbA_1c_ was -3.04 (-2.73 to -3.36); p-value = <0.001). During the treatment, 89.4% of all patients lost weight

Overall, there were no differences between the results in terms of age, gender, race, and the evolution of T2DM diagnosis.

#### Safety

The proportion of patients with adverse events was small. The most frequent adverse event was hypoglycemia in patients using dapagliflozin as an add-on to metformin plus insulin (n = 9/34), and there were two cases of major hypoglycemia. Hypotension and urinary tract infection were infrequent.

## Discussion

This article provides relevant information about population groups not studied in previous research, such as Hispanics and people of African descent. These findings will help clinicians make decisions regarding the treatment of diabetes in countries with a significant population of these ethnic groups. In terms of the other sociodemographic characteristics, the distribution was homogeneous for age and gender among the analyzed groups and the information available in the literature.

Evidence from randomized clinical trials that allows for the evaluation of the behavior of these four combination therapies remains scarce. Recently, Al AdAwi and cols. (15) published a retrospective observational study in which they assessed the efficacy of adding dapagliflozin to four groups of patients, each with dual or triple basal treatment. The results indicated a decrease of 1% to 2.3% in baseline HbA_1c_, which was greater than 9 % in all groups; similarly, baseline glycemia ranged from 36 to 85 mg/dL. In our study, baseline HbA_1c_ reductions ranged from 0.8% to 1.1%, and FPG reductions ranged from 30.9 to 62.4 mg/dL.

Although, benefits were observed in the various clinical outcomes in all four groups, for the group that used dapagliflozin as an add-on to metformin plus glibenclamide, the maximum reduction of HbA_1c_, its stable trend over time, and its results in terms of decrease in FPG and the percentage of patients who reached HbA_1c_ <7% were similar to those reported in series such as Matthaei and cols. (16). However, the reduction in weight was greater in their study (-2.2 to 3.0 kg), which is probably explained by the sulfonylurea used in that research. Other studies (17) show that the use of glimepiride is associated with a decrease in body weight of approximately 3 kg.

The treatment regimen that has received the greatest support in medical literature is the use of dapagliflozin as an add-on to metformin plus saxagliptin. Randomized double-blind clinical trials that compared this triple therapy to dual therapy plus placebo reported a reduction in HbA_1c_ of 0.96% at 24 weeks (18) and 0.74% at 52 weeks (19), with a total reduction between 1.09% (20) and 1.2% (21). These results are similar to ours, as were FPG values reported in previous studies (18–21). Likewise, the proportion of patients with HbA_1c_ <7% reported in other publications ranged from 29% (19) to 47% (20) at 52 weeks and from 30% to 45% at 24 weeks (18), with weight loss that ranged from 1.4 to 2.4 kg in the other series (18–21). These findings are comparable with the present study; in fact, based on our findings, this regimen appears to be the most effective.

The greatest weight loss (between 2.2 and 3.8 kg) was observed in the dapagliflozin add-on to metformin plus exenatide group, which had a sustained linear downward trend. These data are similar to those described in the DURATION-8 study, which showed reductions of 1 to 2.6 kg at week 28 (22) and 0.7 to 2.8 kg at week 52. Their trend was not linear; however, it did stabilize from week 28 to week 52 (23). This can be explained by the effect of GLP1 analogs on weight, as they induced reductions in responders of up to 4.2 kg among diabetics (24), and up to 8 kg in non-diabetics (25). In our study, this group also had a slightly higher reduction in HbA_1c_ and FPG when compared to the data obtained by Jabbour and cols. (23). However, they reported that the percentage of patients with HbA_1c_ <7% reached 53.9% at 28 weeks and 44% at 52 weeks (23), which is a higher proportion than reported in our results.

The highest reduction in HbA1c was seen in the dapagliflozin add-on to metformin plus insulin group, with reductions reported of up to 1.1%, a linear trend until week 24, and a subsequent stabilization at the same value until week 52. Although there are few studies on this combination as a triple or dual therapy, Al AdAwi and cols. included a triple therapy group with insulin, dapagliflozin, and other oral anti-diabetic agents, which reached a maximum HbA_1c_ reduction of 2.3% at week 52 with a continuous linear downward trend (15). This can be explained by the presence of an initial HbA_1c_ value higher than that reported for our patients in this study (10.8% vs. 8.3%). In other series with initial HbA_1c_ close to 10%, there have been decreases of 1.6% reported at 12 weeks (26), and of 0.74% at 52 weeks in the DAISY (Dapagliflozin Added to patients under InSulin therapY) trial (27). Likewise, in subgroup analyses from the DIAMOND study program, Billings and cols. demonstrated that the magnitude of the change in HbA_1c_ was numerically greatest for the highest baseline subgroup (28). Regarding weight, the later study also showed a reduction similar to the present study’s findings. An important point found in the publications is the insulin dose and its reduction when dapagliflozin is started, which varies from 3 units (27) to 19 units (26). Unlike our series, the mean of international units per day was not modified throughout the study, so it did not contribute to the change in HbA_1c_ level.

The reduction in systolic and diastolic BP in all the groups studied was notable, as it ranged from 15.82 to 27.41 mmHg for systolic BP and from 3.97 to 10.86 mmHg for diastolic BP, especially when compared to other series that did not exceed 7 mmHg of reduction in systolic BP values and 2.9 mmHg in diastolic BP values (16–21). This could be explained due to the need to optimize BP control as part of a new model of integrated diabetes care using a multidisciplinary team approach. Our patients received follow-up care in a cardiovascular risk program in which adjustments were made in the antihypertensive therapy (47.31%, 22.76%, and 18.78% at 12, 24 and 52 weeks). Furthermore, a very high initial BP has not been observed in other series, in which mean systolic BP was 130 mmHg (16–21) compared to 148 mmHg in the present study. Publications that assessed the hypotensive effect of dapagliflozin found variations in the decrease in BP, which depends on whether the patient is hypertensive. A decrease ranging from 1.2 to 4.9 mmHg in systolic BP at 12 weeks (27,29) was reported; in addition, among hypertensive patients, a decrease in systolic BP of 3.6 mmHg and a decrease in diastolic BP of 1.2 mmHg at 24 weeks was reported, with decreases of 2.6 and 1.2 mmHg, respectively, among non-hypertensive patients (30). There might be other variables and confounding factors not taken into consideration, such as adjustment of antihypertensive medication during follow-up care performed by specialists from unidentified medical centers.

All the patients received a T2DM diagnosis 4.5 to 7.3 years ago, and no patient reached the glycemic control target with dual therapy. This can be explained by the progressive decline of β-cell function over the years, after the diagnosis of diabetes. Turner and cols. described the difficulty of achieving tight glycemic control on monotherapy during the first 3 years following a diagnosis of diabetes. Likewise, approximately 9 years after receiving the diagnosis, 75% of patients needed multiple therapies to achieve the target (31).

In our study, elderly patients achieved a greater downward trend reduction in HbA_1c_ and systolic BP. These data are similar to those analyzed in the DECLARE-TIMI 58 study, which showed a greater reduction in this age group, although without statistically significant differences (32). There were no differences between sexes, and SGLT-2i has not shown a sex-by-drug interaction, so it is effective and safe regardless of sex (33).

In reference to systolic BP, greater reductions of up to 4 mmHg were reported among individuals of African descent compared to Hispanics. In a randomized, placebo-controlled double-blind study, in black patients with T2DM, SGLT-2i had a fully antihypertensive effect at 6 months, with a reduction up to 13.74 mmHg (34).

We also evaluated antihypertensive treatment. Our results showed a greater downward reduction of as much as 3.6 mmHg when the patients also took a β-blocker or calcium-channel blocker compared to other types of treatments. In a randomized, double-blind, placebo-controlled study, Weber and cols. reported that BP-lowering properties of dapagliflozin were particularly favorable in patients already receiving a β-blocker or calcium-channel blocker (29).

Our results showed that those patients who received angiotensin-converting enzyme inhibitors (ACEI) or angiotensin receptor blockers (ARBs) had a greater downward reduction in HbA1c and weight loss of up to 0.7 Kg compared to those treated with a β-blocker or calcium-channel blocker. A meta-analysis of randomized controlled studies found that the combination therapy with ACEIs or ARBs and SGLT2i in the treatment of T2DM can exert synergistic effects, which may result in complementary pharmacokinetics and pharmacodynamics (35).

Weight loss was not related to a greater reduction in BP. Oliva and cols. evaluated this and suggested that SGLT-2i may have BP-lowering effects beyond weight loss (36).

### Limitations

The study only demonstrated the behavior of the event within a specialized medical center. Although all the patients who participated during the established period met the selection criteria, the population size was small. At the beginning of the study, the groups were not homogeneous, with FPG, as well as the duration of T2DM, with statistically significant differences. This could have contributed to obtaining greater reductions in HBA1c within subgroups with greater baseline glycemic levels. Furthermore, body mass index was not calculated, given the absence of height data, which eliminated the possibility of grouping the patients according to their weight status.

### Strengths

The evaluation was carried out under real conditions with adherent patients who complied with all established controls as directed by the endocrinology unit. To date, there is no information available in the literature where these 4 management proposals are followed up.

### Public Health Impact

Nowadays, it is not just enough to obtain adequate control based on a glucose-centered goal; the intention is to go beyond it and achieve optimal control of cardiovascular risk factors, including weight and blood pressure, generating a positive impact in reducing the risk of complications in the short and long term, which is reflected in a decrease in health costs.

### Recommendations

Taking into account the results obtained here, dapagliflozin could be considered as an intensification alternative in those uncontrolled type 2 diabetic patients with dual therapy that included optimal dose of metformin, glomerular filtration rate ≥ 45 ml/min, who suffer from overweight and uncontrolled arterial hypertension.

In conclusion, the addition of dapagliflozin as an alternative for intensification of dual therapy of uncontrolled type 2 diabetic patients consistently improved, not only FPG and HbA_1c_, but also body weight and blood pressure, with statistically significant results. The combination of dapagliflozin added to metformin plus saxagliptin showed better results, guaranteeing a greater number of patients with HbA_1c_ <7%.
